# Adherence to COVID-19 preventive measures in Sub-Saharan Africa during the 1st year of the pandemic: Pooled analysis of the International Citizen Project on COVID-19 (ICPCovid) surveys

**DOI:** 10.3389/fpubh.2022.1020801

**Published:** 2022-11-08

**Authors:** Leonard Ngarka, Joseph Nelson Siewe Fodjo, Wepnyu Yembe Njamnshi, John D. Ditekemena, Mohammed A. M. Ahmed, Rhoda K. Wanyenze, Janet Dula, Philippe Sessou, Christian T. Happi, John N. Nkengasong, Robert Colebunders, Alfred K. Njamnshi

**Affiliations:** ^1^Brain Research Africa Initiative (BRAIN), Yaoundé, Cameroon; ^2^Department of Neurology, Yaoundé Central Hospital/Neuroscience Laboratory, Faculty of Medicine and Biomedical Sciences, The University of Yaoundé I, Yaoundé, Cameroon; ^3^Global Health Institute, University of Antwerp, Antwerp, Belgium; ^4^Ecole de Santé Publique, Faculté de Médecine, Université de Kinshasa, Kinshasa, Democratic Republic of Congo; ^5^Department of Paediatrics, Faculty of Medicine and Surgery, Mogadishu University, Mogadishu, Somalia; ^6^Department of Paediatric Cardiology, Uganda Heart Institute, Kampala, Uganda; ^7^School of Public Health, College of Health Sciences, Makerere University, Kampala, Uganda; ^8^Programa de Politicas e Sistemas de Saúde, Instituto Nacional de Saúde, Maputo, Mozambique; ^9^Research Unit of Communicable Diseases, Polytechnic School of Abomey-Calavi, University of Abomey-Calavi, Cotonou, Benin; ^10^African Center of Excellence for Genomics of Infectious Disease, Redeemer's University, Ede, Nigeria; ^11^Center for Disease Control and Prevention (CDC) Africa, African Union, Addis Ababa, Ethiopia

**Keywords:** COVID-19, Sub-Saharan Africa, barrier measures, adherence score, prevention

## Abstract

**Introduction:**

While most governments instituted several interventions to stall the spread of COVID-19, little is known regarding the continued observance of the non-pharmaceutical COVID-19 preventive measures particularly in Sub-Saharan Africa (SSA). We investigated adherence to these preventive measures during the initial 6 months of the COVID-19 outbreak in some SSA countries.

**Methods:**

Between March and August 2020, the International Citizen Project on COVID-19 consortium (www.icpcovid.com) conducted online surveys in six SSA countries: Benin, Cameroon, Democratic Republic of Congo, Mozambique, Somalia, and Uganda. A five-point individual adherence score was constituted by scoring respondents' observance of the following measures: mask use, physical distancing, hand hygiene, coughing hygiene, and avoiding to touch one's face. Community behaviors (going to public places, traveling during the pandemic) were also assessed. Data were analyzed in two time periods: Period 1 (March-May) and Period 2 (June-August).

**Results:**

Responses from 26,678 respondents were analyzed (mean age: 31.0 ± 11.1 years; 54.1% males). Mean individual adherence score decreased from 3.80 ± 1.37 during Period 1, to 3.57 ± 1.43 during Period 2; *p* < 0.001. At the community level, public events/places were significantly more attended with increased travels during Period 2 compared to Period 1 (*p* < 0.001). Using linear mixed models, predictors of increased individual adherence included: higher age (Coef = 0.005; 95% CI: 0.003–0.007), female gender (Coef = 0.071; 95% CI: 0.039–0.104), higher educational level (Coef = 0.999; 95% CI: 0.885–1.113), and working in the healthcare sector (Coef = 0.418; 95% CI: 0.380–0.456).

**Conclusion:**

Decreasing adherence to non-pharmaceutical measures over time constitutes a risk for the persistence of COVID-19 in SSA. Younger persons and those with lower education levels constitute target groups for improving adherence to such measures.

## Introduction

The coronavirus disease 2019 (COVID-19) has plagued the global scene for over 2 years. By June 16th 2022, the cumulative number of COVID-19 cases around the world stood at 535,248,141 with 6,313,229 deaths since the initial outbreak in Wuhan—China (1). While Europe and the Americas record the highest number of cases and deaths due to the deadly virus, the African continent accounts for <2% of the global COVID-19 burden (1). Initially it was predicted that the COVID-19 death toll would be enormous in Africa due to fragile health systems, precarious living conditions, and anticipated difficulties in observing the non-pharmaceutical preventive measures (2, 3). However, SSA cumulates fewer COVID-19 cases and deaths compared to other regions of the world. Among other reasons, the leading explanation for this relatively low COVID-19 numbers in SSA seems to be the demographic structure with several youths who are less susceptible to develop severe disease (4). Additionally, according to a recent genetic study, ACE2 genes (incriminated in COVID-19 physiopathology) show a rare variation among persons of African origin which may explain why most SSA populations are only mildly affected by the disease (5).

Most SSA countries reported their first case of COVID-19 in March 2020, justifying the World Health Organization's timing in recognizing COVID-19 as a pandemic during that same month (6). Consequently, many nations adopted unprecedented measures to limit viral transmission; such strategies included closure of international borders, closure of schools, and working from home when possible (7). The fact that the population's adherence to these preventive measures was not systematically monitored prompted the initiation of online surveys across several low- and middle-income countries *via* the International Citizen Project on COVID-19 (ICPCovid) (8).

Considering the unforeseen persistence of the pandemic during several years, it expected that adherence trends will vary over time. Moreover, the introduction of pharmaceutical measures against COVID-19 (for example, vaccines) may significantly impact adherence to the non-pharmaceutical strategies that were hitherto prioritized. Thus, understanding the determinants of adherence prior to COVID-19 vaccine deployment would inform public health authorities in SSA on the appropriate strategies to achieve high adherence to non-pharmaceutical measures in the general population during public health emergencies such as COVID-19.in pandemic. In the present paper, we sought to pool together adherence data from six ICPCovid countries in SSA to increase sample size and arrive at conclusions that hopefully would be relevant for most SSA settings. We specifically aimed to analyze the trends in adherence to COVID-19 preventive measures as well as the evolution of the COVID-19 prevalence during the first 6 months of the outbreak in the participating countries.

## Materials and methods

### Data sources

The data analyzed in this study were obtained during online surveys conducted by the ICPCovid consortium in six SSA countries between March and August 2020 (first 6 months of the outbreak in these SSA countries). Participating countries included: Benin, Cameroon, Democratic Republic of Congo (DRC), Mozambique, Somalia, and Uganda. The datasets used in this study are from (9–14).

### Study procedures

In each country, investigators adapted the template ICPCovid questionnaire to the context of their countries and translated it to the national language(s). Thereafter the survey web-link was widely disseminated *via* social media and other platforms to invite consenting participants to fill in their responses through smartphones, tablets or computers. Data were collected on socio-demographic characteristics, as well as the self-reported experience of at least one of the following non-specific flu-like symptoms which constitute the COVID-19 clinical definition (15) during the past 2 weeks: fever, headaches, cough, sore throat, coryza, anosmia, ageusia, shortness of breath, myalgia, fatigue, nausea, or vomiting. The reported symptoms were used to identify participants who met the clinical criteria for suspected COVID-19 based on recommendations from the World Health Organization (WHO) (15).

To assess respondents' preventive behaviors during the pandemic, questions were also asked regarding observance of individual COVID-19 preventive measures (mask use, physical distancing, hand hygiene i.e., regular hand washing with soap and/or use of alcohol-based hand gel, not touching one's face, and covering the mouth when coughing/sneezing). as Adherence to community preventive measures against COVID-19 were also investigated; these included: having attended a gathering with more than 10 persons, having gone to a public gym/beauty center including hair/barbing saloon, been to a market, or having traveled, all within the past 7 days. All responses were anonymously submitted to the secure ICPCovid server (hosted in in Belgium), where they were stored until data extraction for analysis.

### Data analysis

Collected data were exported to Microsoft Excel 2016 spreadsheets for cleaning and later transferred to R version 4.0.2 for analysis. Continuous variables (age and adherence score) were summarized as means and standard deviation (SD). Since the two continuous variables failed the Kolgomorov-Smirnov normality test, they were compared across groups using a Mann-Whitney *U*-test or Kruskal Wallis test as appropriate. Meanwhile, categorical variables (gender, educational level, marital status, profession, residential setting, healthcare worker/student, and adherence to individual preventive measures yes vs. no) were expressed as percentages, and comparisons done using the Chi-square test or Fisher exact test as appropriate. Adherence was measured by attributing a score of one when the respondent observed any of the preventive measures, and zero otherwise. A composite score for individual adherence was constructed by summing up scores from the five individual measures, since this combination of questions yielded the best performance (highest value for Cronbach alpha). In case of missing values for any of the above-mentioned individual preventive measures, the “partial” adherence scores were standardized to a 5-point scale using the formula below and rounded to the nearest whole number:

$$\begin{array}{ll} Adjusted\;Score\;\left(on\;5\right)\\ = & \frac{Total\;score\;for\;“n”\;preventive\;measures}{n}\times 5 \end{array}$$

To investigate whether the level of adherence correlated with the prevalence of flu-like symptoms reported on a weekly basis in our study population, we constituted weekly clusters by grouping all data received during a given week in each country. Given that the reported flu-like symptoms had been experienced within the past 2 weeks, we used the rolling mean prevalence of suspected COVID-19 (considering two consecutive weeks: week *n-1* and week *n*) for this analysis. Only weeks with ≥15 responses in the country-specific datasets were included when constituting the weekly clusters.

The internal consistency of the 5-item adherence score on the overall dataset was assessed using the Cronbach alpha, while its dimensionality was investigated using exploratory factor analysis (EFA). Determinants of individual adherence to COVID-19 preventive measures were investigated *via* a generalized linear mixed model using the 5-point adherence score as the dependent variable. The model was constructed using the lmer function (package: “lme4”) in the software R. The variable “country of residence” was introduced in the random part of the model, while all other variables were fixed covariates. Covariates for the final model were selected based on a *p*-value < 0.2 in univariate analysis. However, the variable “marital status” was excluded from the model due to several missing values (>4,000), despite the significant difference observed during the descriptive and univariate analysis. All *p*-values < 0.05 were considered statistically significant.

Pooled analysis of multi-country adherence scores was done using the software RevMan version 5.3. Surveys were sub-grouped based on the period during which the data was collected: Period 1 (early phase) included surveys that were conducted within the first 3 months March, April, May 2020; and Period 2 (later phase) for surveys conducted during the three remaining months (June, July, August 2020). To investigate whether pooled adherence scores varied by period, sub-group analysis was performed using a random effects model and a forest plot was generated.

### Ethical considerations

The online ICPCovid platform used to conduct the surveys was approved by the Ethics Committee of the University of Antwerp, Belgium (Ref: 20/13/148). Additionally, the respective national ethical committees in each participating country provided their approval (see Author Statements section for details). Only responses from participants aged 18 years and above who provided an e-consent were retained for analysis. All data were collected anonymously and treated with absolute confidentiality.

## Results

### Participant characteristics

Data from a total of 26,678 respondents from the six participating countries were analyzed (overall mean age: 31.0 ± 11.1 years, 54.1% males). Except for Cameroon which had no data for Period 1 and Uganda which had no data for Period 2, all other countries provided adherence data for both study periods. Table 1 presents the socio-demographic characteristics of the participants grouped by country and study period. For both Period 1 and Period 2, most participants resided in urban settings (68.1 and 74.8%, respectively) and had attained a university level of education (74.3 and 64.2%, respectively). There were more participants who reported at least one flu-like symptom during Period 2 (36.5%) than in Period 1 (20.1%); *p* < 0.001. Furthermore, the overall prevalence of suspected COVID-19 cases in our study population significantly increased from 8.7% in Period 1 to 9.8% in Period 2; *p* = 0.003. Of note, detailed flu-like symptoms were not available in the Ugandan survey hence it was not possible to identify suspected COVID-19 cases.

**Table 1:** Participant characteristics by study period.

**Country**	**Benin**		**Cameroon** ^a^		**Dem. Rep. Congo**		**Mozambique**		**Somalia**		**Uganda**	
**period**	**Period 1**	**Period 2**	**Period 1**	**Period 2**	**Period 1**	**Period 2**	**Period 1**	**Period 2**	**Period 1**	**Period 2**	**Period 1**	**Period 2**
	***N* = 462**	***N* = 507**	***N* = NA**	***N* = 3,047**	***N* = 3,380**	***N* = 3,850**	***N* = 3,770**	***N* = 1,150**	***N* = 4,116**	***N* = 4,684**	***N* = 1,712**	***N* = NA**
Age in years: mean (SD)	28.9 (9.1)	30.9 (9.0)	NA	33.5 (10.9)	36.3 (12.3)	35.6 (11.3)	34.7 (10.6)	33.6 (9.2)	22.9 (5.4)	23.7 (6.0)	36.1 (10.4)	NA
Male gender: *n* (%)	347 (75.1)	370 (73.0)	NA	2,263 (74.3)	1,158 (34.3)	1,175 (30.5)	2,174 (57.7)	674 (58.6)	2,490 (60.5)	2,768 (59.1)	1,005 (58.7)	NA
**Marital status:** ***n*** **(%)**			NA									NA
Single	301 (65.2)	296 (58.4)		1,587 (52.1)	851 (25.2)	924 (24.0)	1,681 (44.6)	546 (47.5)	3,537 (85.9)	3,730 (79.6)	666 (38.9)	
Cohabitation	51 (11.0)	69 (13.6)		320 (10.5)	297 (8.79)	519 (13.5)	655 (17.4)	217 (18.9)	42 (1.0)	68 (1.5)	246 (14.4)	
Legally married	107 (23.2)	137 (27.0)		1,072 (35.2)	2,067 (61.2)	2,205 (57.3)	1,296 (34.4)	357 (31.0)	497 (12.1)	770 (16.4)	752 (43.9)	
Divorced/widow(er)	3 (0.6)	5 (1.0)		68 (2.2)	165 (4.9)	202 (5.2)	138 (3.6)	30 (2.6)	40 (1.0)	116 (2.4)	48 (2.8)	
**Highest educational level:** ***n*** **(%)**			NA									NA
Primary	0 (0.0)	8 (1.6)		34 (1.1)	160 (4.7)	242 (6.3)	17 (0.5)	6 (0.5)	20 (0.5)	130 (2.8)	1 (0.1)	
Secondary	48 (10.4)	50 (9.9)		808 (26.5)	1,729 (51.2)	2,447 (63.6)	1,151 (30.5)	332 (28.9)	284 (6.9)	561 (12.0)	56 (3.3)	
Undergraduate	228 (49.4)	184 (36.3)		1,226 (40.2)	1,272 (37.6)	1,022 (26.5)	2,596 (68.9)	811 (70.5)	3,424 (83.2)	3,495 (74.6)	860 (50.2)	
Postgraduate	186 (40.3)	265 (52.3)		979 (32.1)	219 (6.5)	139 (3.6)	6 (0.2)	1 (0.1)	388 (9.4)	498 (10.6)	795 (46.4)	
**Residential setting:** ***n*** **(%)**			NA									NA
Rural	55 (11.9)	64 (12.6)		274 (9.0)	209 (6.2)	31 (0.8)	549 (14.6)	187 (16.3)	47 (1.1)	125 (2.7)	188 (11.0)	
Sub-urban	196 (42.4)	188 (37.1)		563 (18.5)	461 (13.7)	898 (23.3)	1,353 (35.9)	434 (37.7)	202 (4.9)	365 (7.8)	1,016 (59.3)	
Urban	211 (45.7)	255 (50.3)		2,210 (72.5)	2,691 (80.1)	2,921 (75.9)	1,868 (49.5)	529 (46.0)	3,867 (94.0)	4,194 (89.5)	508 (29.7)	
**Profession**			NA									NA
Student	219 (47.4)	173 (34.1)		715 (23.5)	424 (12.5)	565 (14.7)	497 (13.2)	165 (14.3)	2,923 (71.0)	3,057 (65.3)	200 (11.7)	
Unemployed	38 (8.23)	55 (10.8)		608 (20.0)	1,364 (40.4)	1,458 (37.9)	353 (9.4)	78 (6.8)	359 (8.7)	594 (12.7)	124 (7.2)	
Self-employed	38 (8.2)	56 (11.0)		345 (11.3)	580 (17.2)	884 (23.0)	105 (2.8)	22 (1.9)	197 (4.8)	361 (7.7)	283 (16.5)	
Private sector employee	64 (13.9)	106 (20.9)		751 (24.6)	527 (15.6)	451 (11.7)	1,554 (41.2)	355 (30.9)	562 (13.7)	559 (11.9)	729 (42.6)	
Government employee	103 (22.3)	117 (23.1)		628 (20.6)	485 (14.3)	492 (12.8)	1,261 (33.4)	530 (46.1)	75 (1.8)	113 (2.4)	376 (22.0)	
Healthcare student /worker: *n* (%)	170 (36.8)	147 (29.0)	NA	361 (11.8)	469 (13.9)	439 (11.4)	910 (24.1)	446 (38.8)	1,084 (26.3)	1,581 (33.8)	616 (36.0)	NA
Lives alone in household: *n* (%)	54 (11.7)	69 (13.6%)	NA	582 (19.1)	169 (5.0)	0 (0)	267 (7.1)	88 (7.7)	245 (5.9)	278 (5.9)	246 (14.4)	NA
Suspected cases of COVID-19: *n* (%)	47 (10.2%)	70 (13.8%)	NA	408 (13.4%)	349 (10.3%)	270 (7.0%)	223 (5.9%)	70 (6.1%)	406 (9.9%)	484 (10.3%)	NA	NA

a Although data from Cameroon was collected from June—December 2020, only responses collected between June—August 2020 were analyzed in this study to respect the time bounds of Period 2.

SD, Standard deviation; NA, Not available.

### Adherence to individual COVID-19 preventive measures

**Figure 1:**
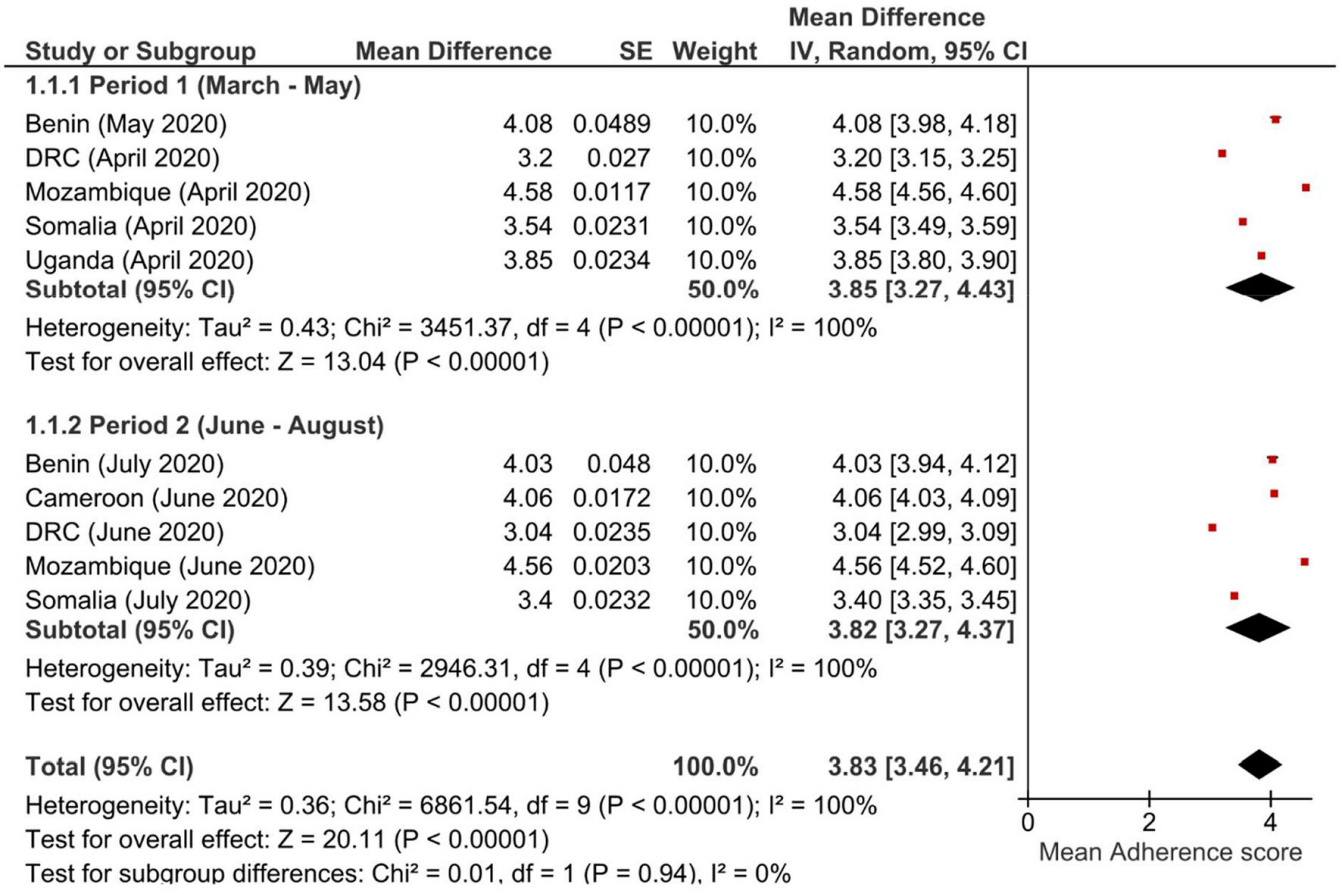
Forest plot of adherence scores by study period.

An overall decrease in the adherence to preventive measures was observed between Period 1 and Period 2 (Table 2). Pooled analysis by Period also showed a similar trend, with adherence scores decreasing from 3.85 in Period 1, to 3.82 in Period 2, albeit being non-significant statistically (Figure 1); of note, a high degree of heterogeneity was observed in the pooled analysis. Mean adherence score was higher among men (3.76) compared to women (3.58), *p* < 0.001; higher among participants in the healthcare sector (4.11) compared to other respondents (3.55), *p* < 0.001; higher among the more educated (postgraduate > undergraduate > secondary > primary), *p* < 0.001; and higher among rural residents (rural > suburban > urban), *p* < 0.001.

**Table 2:** Adherence to preventive measures during the study periods.

	**Period 1**	**Period 2**	***P*-value**
	**(*N* = 13,440)**	**(*N* = 13,238)**	
**Overall adherence to individual preventive measures:** ***n*** **(%)**			
Use face mask in public	8,063 (60.6%)	9,666 (73.9%)	< 0.001
Observe physical distancing	9,740 (72.5%)	7,891 (59.6%)	< 0.001
Hand hygiene (regular hand washing with soap and/or use of alcohol-based hand gel)	12,434 (92.5%)	11,521 (87.0%)	< 0.001
Avoiding to touch face	9,468 (70.4%)	8,041 (60.7%)	< 0.001
Covering the mouth when coughing/sneezing	11,334 (84.3%)	10,074 (76.1%)	< 0.001
**Adherence score to individual preventive measures per country: mean (SD)**			
Benin	4.08 (1.05)	4.03 (1.08)	0.439
Cameroon	NA	4.06 (0.95)	NA
Democratic Republic of Congo	3.20 (1.57)	3.04 (1.46)	< 0.001
Mozambique	4.58 (0.72)	4.56 (0.69)	0.152
Somalia	3.54 (1.48)	3.40 (1.59)	< 0.001
Uganda	3.85 (0.97)	NA	NA
Overall	3.80 (1.37)	3.57 (1.43)	< 0.001

SD, Standard deviation; NA, Not available.

Regarding the internal consistency of the 5-item adherence score, the overall Cronbach alpha for the entire dataset was 0.669. Upon stratifying the data by country, disparities in internal consistency were observed; the following Cronbach alpha values were obtained: 0.521 for Benin (*n* = 969), 0.263 for Cameroon (*n* = 3,047), 0.661 for DRC (*n* = 7,230), 0.360 for Mozambique (*n* = 4,920), 0.725 for Somalia (*n* = 8,800), and 0.447 for Uganda (*n* = 1,712). Exploratory factor analysis (EFA) revealed that except for “mask use,” all other items loaded on one factor with factor loadings > 0.5. Mask use had a high loading (0.786) on a different factor (Supplementary Appendix 1).

### Adherence to community COVID-19 preventive measures

There was an increase in non-observance of the community measures over time in our study population. Combined data from all participating countries show that compared to Period 1, Period 2 was characterized by more participants reporting that: they attended gatherings with ≥10 persons (from 12.7 to 39.4%, *p* < 0.001); they have been to public gym (from 3.5 to 12.1%, *p* < 0.001); they have been to a beauty center, hair/barbing saloon (from 22.4 to 35.8%, *p* < 0.001); they have been to a market (from 55.7 to 63.3%, *p* < 0.001); they traveled outside of their town of residence (from 8.4 to 18.1%, *p* < 0.001).

**Figure 2:**
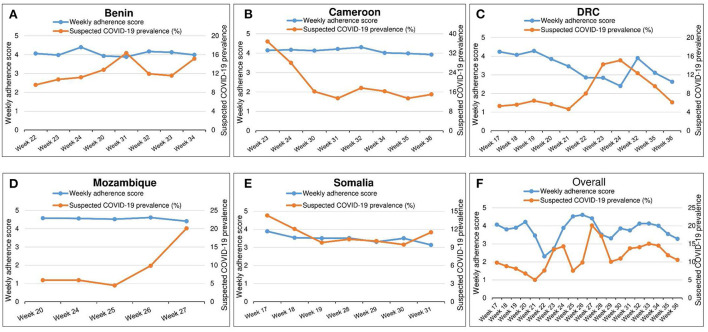
**(A–F)** Evolution of weekly adherence scores (for week *n*) and the rolling mean week-specific prevalence of suspected COVID-19 (for week *n-1* and week *n*). Uganda is not included in this analysis because data on flu-like symptoms was not collected during the Ugandan ICPCovid survey.

Regarding the analysis of the weekly adherence scores in relation to the rolling mean prevalence of suspected COVID-19, there was no correlation (Spearman rho = −0.035, *p*-value = 0.831). Figure 2 graphically presents the evolution of weekly adherence scores and suspected COVID-19 prevalence by country and by week (week numbers are given as per the 2020 yearly calendar). Based on rough estimates derived from the shape of the graphs, it appears that to produce a noticeable curb in the burden of COVID-19-like symptoms, the adherence scores must exceed the threshold value of four.

### Determinants of adherence score

The multivariable model showed that data collected during Period 2 was associated with lower adherence scores. Additionally, having experienced any flu symptoms during the past 2 weeks, residing in urban/sub-urban settings, and being unemployed/self-employed were also associated with reduced adherence to the preventive measures. Increasing age, female gender, higher educational level and working in the healthcare sector were all associated with increased adherence scores (Table 3). The random part of the model, represented by the variable “country of residence,” accounted for 11.6% of the variance in adherence score observed in our study population.

**Table 3:** Multivariable model investigating determinants of adherence score (*N* = 26,575).

**Covariates**	**Adjusted regression coefficient (95% CI)**	***P*-value**
Age (in years)	0.005 [0.003, 0.007]	<0.001
Female gender	0.071 [0.039, 0.104]	<0.001
Study period 2	−0.057 [−0.092, −0.022]	0.001
**Education**		
Primary	Reference	
Secondary	0.595 [0.490, 0.699]	<0.001
Undergraduate	0.942 [0.837, 1.049]	<0.001
Postgraduate	0.999 [0.885, 1.113]	<0.001
**Profession**		
Student	Reference	
Unemployed	−0.261 [−0.312, −0.209]	<0.001
Self-employed	−0.167 [−0.227, −0.108]	<0.001
Private sector employee	−0.026 [−0.076, 0.024]	0.312
Government employee	−0.039 [−0.097, 0.019]	0.191
Healthcare worker or student	0.418 [0.380, 0.456]	<0.001
Flu symptom(s) during past 14 days	−0.217 [−0.252, −0.183]	<0.001
**Residential setting**		
Rural	Reference	
Sub-Urban	−0.430 [−0.499, −0.361]	<0.001
Urban	−0.224 [−0.290, −0.159]	<0.001
**Random effects**		
Variance of the variable “country of residence”	0.2095	
Residual variance	1.5913	
Number of groups	6	

## Discussion

In this multi-country study, we examined adherence trends with respect to COVID-19 non-pharmaceutical preventive measures implemented in six SSA countries, and the concomitant evolution of the burden of COVID-19-like illness among the participants in these countries. We proposed a scoring system to best evaluate the population's adherence to these COVID-19 preventive measures. The adherence score showed an optimal overall Cronbach alpha value of 0.669 when the five individual preventive measures recommended by the WHO (16) were retained. Given that Cronbach alpha values for the 5-item adherence score varied greatly by country, and with the high heterogeneity observed during the pooled analysis, it is evident that the proposed scoring system still requires context-specific improvements and is hardly generalizable to different SSA countries.

We noted a significant drop in overall individual adherence over time, associated with an evident rise in the prevalence of COVID-19-like illness, which may have contributed to the resurgence (second wave) of the COVID-19 cases observed in Africa at the wake of 2021. Indeed, during the early months of the pandemic, many people were initially conscious and ready to adhere probably due to all the sensitizations on COVID-19 and its reported deleterious effects on human health. When individuals in the participating countries progressively noticed that they were not being decimated by the pandemic as per the predictions, coupled with the economic hardships engendered by the lockdown measures (17), they might have become more relaxed in observing the preventive measures just a few months after their implementation. Furthermore, governments might have become less strict about reinforcing the instituted measures after the first epidemic peak had passed in these countries. Although our web-based approach introduced a selection bias, the overall findings suggest that adherence to COVID-19 preventive measures gradually declined. This declining adherence possibly fostered the rise in COVID-19 incidence observed in numerous African countries (18, 19). Our findings corroborate with the occurrence of a second wave of COVID-19 in several African countries during the second half of the year 2020 (18, 19).

Although the overall drop in mean adherence score between Period 1 and Period 2 was significant, there were inter-country disparities. Countries such as the DRC and Somalia recorded a major drop in adherence scores, while Period 1 and Period 2 adherence scores were not significantly different for Benin and Mozambique. In Mozambique, the majority of the study population worked from home during both periods of the study, suggesting that community preventive measures were strictly observed by public and private institutions, possibly enhancing adherence to individual measures as well (11). Meanwhile in Benin, the government did not opt for a radical lockdown but rather insisted on more friendly barrier measures while allowing free movement of people (9); we presume that complying with this non-stringent strategy was not difficult, and therefore adherence scores remained more or less constant throughout the study duration in Benin. We further observed a rise in face mask usage during Period 2, and a decreased observance of other COVID-19 preventive measures, including physical distancing. This could be due to the fact that in SSA settings, distancing is not always easy in densely populated urban milieus, especially for individuals who work in the informal sector. Therefore, they tend to compensate the non-observance of distancing by increasing their face mask use in order to continue their professional duties amidst the pandemic. Additionally, face masks were in very limited supply during the early months of the pandemic (20), and this could explain why fewer persons wore masks in Period 1. Furthermore, the fact that “mask use” loaded on a different factor than other COVID-19 preventive measures during EFA suggests that it is perceived as a stand-alone intervention, with its own unique adherence trends. The increasing use of face masks is an encouraging finding, considering a recent meta-analysis which revealed that the protective effect of masking (using N95 or surgical masks) was comparable to that of observing distancing >1 meter (21). However, it is still necessary to promote physical distancing as this is a proven method to quell the COVID-19 pandemic by limiting inter-personal contacts (22).

Our study found that female gender was associated with an increase in individual adherence scores. Previous research in other SSA countries equally reported a more favorable attitude toward adherence to COVID-19 preventive measures in women (23, 24). Other studies have demonstrated that the psychological distress resulting from the COVID-19 pandemic and associated restrictions tend to affect females more than males (25), and that women are more concerned about their health and that of their family vis-à-vis COVID-19 (26). Additionally, males are more prone to non-adherence as they seek to provide for their families amidst the socio-economic hardship brought about by the pandemic, at the expense of observing the strict measures instituted to reduce viral transmission (27, 28).

From this study, we surmise that achieving an individual adherence score of at least four on five would noticeably curb the incidence of COVID-19-like illness in affected communities. This suggests that better adherence to the prescribed preventive measures may indeed contribute to limiting the risk of contracting COVID-19. Considering the modest internal consistency of the 5-item adherence score, we admit that it can hardly be used to accurately explain the variation in COVID-19 burden and transmission in different countries. Developing an ideal adherence score that would reflect the COVID-19 dynamics in a given community would indeed be a daunting task for a number of reasons: Firstly, fomite transmission of the coronavirus is currently being questioned (29, 30) thereby oppugning the role of handwashing or avoiding to touch one's face in preventing infection. Secondly, household transmission was identified as a major drive behind successive COVID-19 waves, notably in South Africa (31). High transmission within a given bubble highlights the difficulty of relying on community preventive measures to contain the infection. This certainly warrants further research.

We noted increased adherence scores among respondents who reported to be health personnel. As those at the frontline in the fight against the COVID-19 pandemic, health personnel are in constant contact with infected patients, including the hospitalized severe cases. Furthermore, persons in healthcare are more knowledgeable on COVID-19 scientific information updates with regards to prevention and treatment strategies and are best informed on the need for the strict respect of preventive measures. Hence, the compliance of healthcare workers could be leveraged to revamp adherence in the general population by using the health personnel as effective and trusted channels of communication and sensitization (10–13). We also observed that the participant's profession played a role in influencing adherence, similar to what was reported by another study in Ethiopia, where government and private organization workers were more likely to observe the preventive measures (32). In our own study, being unemployed or self-employed was associated with a lower adherence score with a trend of increasing adherence scores among those working in private or government institutions. One possible reason for this observation could be the reinforcement of preventive measures by private institutions and public workplaces *via* the provision of hand-washing stations, hand sanitizers, personal protective equipment, face masks, and imposing mandatory physical distancing as required by the government (21).

After more than 2 years into the COVID-19 pandemic, the implementation of measures to minimize transmission and curb COVID-19 incidence and prevalence has been shown to be effective when strictly respected. Currently, vaccines have become the centerpiece in the fight against COVID-19 (33). Available evidence reveals that although the approved COVID-19 vaccines are indeed effective at preventing the disease, the acquired immunity wanes with time especially when the newer variants of the virus are concerned (34). As such, vaccinated persons may still get infected and transmit the virus albeit to a lesser extent (35). While the non-pharmacological preventive measures might not produce the expected public health infection containment benefit (36), they are still crucial in limiting inter-person COVID-19 transmission even in the post-vaccination era.

## Limitations of the study

Since our data were collected *via* an online approach, we recognize that only a particular group of individuals could participate (mostly educated persons, with good internet access and residing in urban settings) thereby introducing both selection and social desirability bias. However, although the findings are hardly generalizable to the general population of the included countries, they do highlight factors associated with adherence to the COVID-19 non-pharmaceutical preventive measures which are probably ubiquitous across most SSA countries. While the level of adherence to the preventive measures may be different among persons who did not participate in the survey (potentially worse as less educated persons, who tend to be less adherent, were under-represented in our study population), the adherence trends in the general population would most likely be similar to what we report in this paper. The proposed adherence score, constituted by purposeful selection of a few variables, may not fully capture the respondents' behavior vis-à-vis the national COVID-19 preventive measures in their country as this greatly varies in the different countries. Therefore, it may be worth investigating adherence scores that include other (combinations of) non-pharmaceutical measures against COVID-19 in the different countries, and that can also account for country-specific variables like number of COVID-19 tests done, strictness of confinement, and vaccination coverage.

We also admit that the WHO clinical case definition which we used to identify suspected cases of COVID-19 is far from accurate in estimating the real number of infected persons, as it does not take into account asymptomatic carriers whose impact in the COVID-19 transmission chain is undeniable (31). Indeed, studies have shown that COVID-19 burden estimates can vary greatly depending on the case definition used (37). With the onset of new COVID-19 waves even in communities with high adherence to non-pharmaceutical interventions (38), it remains unclear whether the latter, if not supplemented with other strategies, would be able to curb the COVID-19 burden in the long run. An additional limitation was the fact that we were unable to assess the external validity of our 5-item score; we therefore recommend that in future adherence surveys, specific questions should be asked that would provide relevant data for estimating the generalizability of a proposed score in a given country or community (39).

## Conclusion

In conclusion, we report a decreasing trend in adherence to COVID-19 preventive measures in SSA countries. This represents a serious risk for resurgence of the virus as evidenced by second, third, and even fourth waves of COVID-19 in some African countries (40). While the advances in pharmaceutical tools against COVID-19 are laudable, proven non-pharmaceutical options can be still be adapted and implemented to ensure a persistent downward trajectory of COVID-19 incidence. Particularly, younger persons with lower education levels should be targeted to improve compliance to preventive measures in SSA settings. Finally, mass vaccination campaigns around the globe should not demotivate observance of non-pharmaceutical measures. Rather, the populations should be sensitized on the synergistic effects of vaccination and continued adherence to the preventive measures to contain the ongoing pandemic sooner rather than later.

## Data availability statement

Publicly available datasets were analyzed in this study. This data can be found at: Data available upon reasonable request from the ICPCovid consortium: icpcovid@uantwerpen.be.

## Author contributions

AN, LN, JS, WN, and RC conceived and designed this study. JDi, MA, RW, JDu, PS, and AN supervised data collection. JS cleaned and analyzed the data. LN, JS, and AN wrote the initial draft of the manuscript. All authors contributed to the editing of the initial draft and approved the final manuscript.

## Funding

The establishment of the ICPCovid website to conduct the surveys was supported by a grant from the European Research Council (ERC 671055). No funding source supported the drafting and dissemination of this scientific article.

## Conflict of interest

The authors declare that the research was conducted in the absence of any commercial or financial relationships that could be construed as a potential conflict of interest.

## Publisher's note

All claims expressed in this article are solely those of the authors and do not necessarily represent those of their affiliated organizations, or those of the publisher, the editors and the reviewers. Any product that may be evaluated in this article, or claim that may be made by its manufacturer, is not guaranteed or endorsed by the publisher.
