# Supplementary material for: Adherence to COVID-19 preventive measures in Sub-Saharan Africa during the 1st year of the pandemic: Pooled analysis of the International Citizen Project on COVID-19 (ICPCovid) surveys
Source: Front Public Health. 2022 Nov 8;10:1020801. doi: 10.3389/fpubh.2022.1020801 (PMC9679527; doi:10.3389/fpubh.2022.1020801)
Supplement: Supplementary file 1 [file Data_Sheet_1.PDF]

# Supplementary Appendix 1

## Exploratory Factor Analysis on the 5-point Adherence Score

| | Factor 1 | Factor 2 |
|---|---|---|
| Standard deviation | 1.806 | 1.148 |
| Proportion of variance | 0.361 | 0.230 |
| Cumulative proportion | 0.361 | 0.591 |

## Loadings on different factors

| | Factor 1 | Factor 2 |
|---|---|---|
| Mask use | 0.247 | 0.786 |
| Physical distancing | 0.611 | 0.458 |
| Hand hygiene | 0.549 | 0.453 |
| Avoid touching face | 0.795 | 0.208 |
| Covering the mouth when coughing or sneezing | 0.663 | 0.268 |
